# Effect of glutathione, ascorbic acid, and their combination on the quality and fertility of cryopreserved spermatozoa of Black Bengal buck

**DOI:** 10.1016/j.vas.2025.100549

**Published:** 2025-11-22

**Authors:** Md. Abul Bashar, Mst. Mahomudha Akhtar, Shahanaj Ferdousi Shejuty, Gautam Kumar Deb, Sadek Ahmed, M. A. M. Yahia Khandoker

**Affiliations:** a Department of Animal Breeding and Genetics, Bangladesh Agricultural University, Mymensingh 2202, Bangladesh; b Biotechnology Division, Bangladesh Livestock Research Institute, Savar, Dhaka 1341, Bangladesh; c Goat Production Research Division, Bangladesh Livestock Research Institute, Savar, Dhaka 1341, Bangladesh

**Keywords:** Black Bengal buck, Antioxidants, Cryopreservation, Sperm quality, Artificial insemination, Fertility

## Abstract

This study investigated the effect of the addition of reduced glutathione (GSH), l-ascorbic acid (AA), and their combination (GSH+AA) to the semen extender on the semen quality and fertility of post-thaw spermatozoa of Black Bengal bucks. Ejaculates (*n* = 30) collected from five Black Bengal bucks were evaluated, pooled (37 °C), and divided into four treatment groups: a control group (no antioxidants), a GSH group (control + 1 mM GSH), an AA group (control + 8 mM AA), and a combined group (control + 1 mM GSH and 8 mM AA). The semen samples were then filled into 0.25 mL French straws and cryopreserved following a standard cryopreservation method. Supplementation of GSH (1 mM) and AA (8 mM) significantly (*P* < 0.05) improved total motility, progressive motility, normal morphology (normal fraction, distal droplet, and bent tail), and kinematic parameters (average path velocity, straight-line velocity, curvilinear velocity, and beat cross frequency) of the spermatozoa. However, the maximum improvement was observed with the combined group. In terms of malondialdehyde (MDA) levels and hydrogen peroxide (H_2_O_2_) status, the values of MDA and H_2_O_2_ were reduced significantly (*P* < 0.05) in the GSH, AA, and GSH+AA groups compared to the control group. The fertilization capacity of the cryopreserved spermatozoa was significantly higher (*P* < 0.05) in the antioxidant-treated groups compared to the control group. In conclusion, supplementation of GSH and AA in freezing medium has a synergistic and beneficial effect on the quality and fertility of cryopreserved spermatozoa of Black Bengal buck.

## Highlights

• Supplementing extenders with 1 mM GSH and 8 mM AA improved post-thaw sperm quality.
• MDA and H₂O₂ levels were significantly reduced in antioxidant-treated groups.
• Fertility of spermatozoa improved with antioxidant supplementation.
• GSH and AA have a synergistic effect on sperm cryotolerance and fertility.

## Graphical abstract

**Figure:**
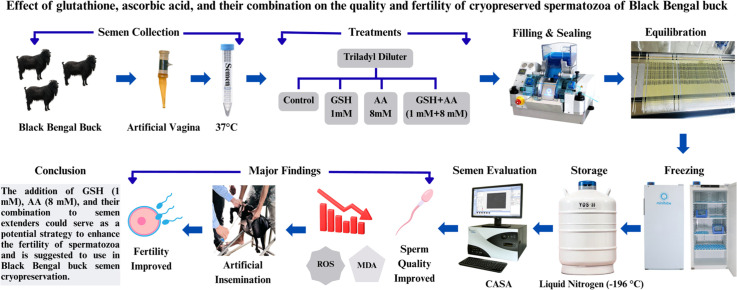

## 1 Introduction

Goats are widely recognized across tropical and developing nations for their valuable contributions to meat, fiber, and skin production. In Bangladesh, goat farming represents an essential and fundamental part of the country's rural farming system. Bangladesh possesses only one goat breed, popularly known as the Black Bengal goat, which holds the second position in the livestock sector in terms of meat and skin production. This goat is widely recognized for its prolificacy, fertility, fecundity, and adaptability to local environments (Hasan et al., 2015; Husain et al., 1996). However, there is a severe shortage of Black Bengal breeding bucks, which are essential for the successful propagation of this breed (Khandoker et al., 2011). This shortage is primarily caused by the common practice of castrating most male kids at an early age for economic benefits and social reasons, which limits the availability of mature males for breeding purposes (Khandoker et al., 2007). This situation often leads to the use of the same bucks over an extended period in the same geographic area, which increases inbreeding practices among the population (Khandoker et al., 2011). Cryopreservation of semen and implementation of artificial insemination (AI) would enable the utilization of superior bucks across a wider geographic area and help address existing challenges within a short timeframe.

AI is a widely used biotechnological tool in animal breeding due to its cost-effectiveness, simplicity, and greater efficiency compared to embryo transfer for the rapid dissemination of elite livestock genetics (Ren et al., 2019). Semen cryopreservation is the crucial step in AI and facilitates the long-term preservation of spermatozoa, maintains fertilization potential, enables long-distance distribution of genetic materials, and leads to greater utilization of genetically superior males (Casali et al., 2017; Zhao et al., 2009). It is a useful technology in the management of reproduction in goat production (Barbas & Mascarenhas, 2009). However, cryopreservation of spermatozoa includes a series of processes that impose physical, chemical, and oxidative stress on spermatozoa, which results in alterations in the plasma membrane structure and acrosome status (Banday et al., 2017). These alterations collectively reduce the fertilizing capacity of spermatozoa after cryopreservation (Watson, 2000). Oxidative stress is considered the primary factor responsible for these detrimental effects on sperm quality due to the activities of reactive oxygen species (ROS), which elevate the lipid peroxidation (LPO) rate during the storage period (Aitken, 2017). Mammalian sperm are particularly vulnerable to oxidative damage due to their limited cytoplasmic content and high levels of polyunsaturated fatty acids in their membranes compared to other species (Özer Kaya et al., 2018). Generally, both seminal plasma and spermatozoa possess intrinsic antioxidant defense mechanisms to neutralize the harmful effects of ROS (Gadea et al., 2011). However, the protective efficacy of these antioxidants against oxidative stress is significantly reduced after the dilution of semen before cryopreservation (Peris-Frau et al., 2020). Therefore, there is growing interest in utilizing exogenous antioxidants to uphold ROS balance and protect spermatozoa from the adverse effects of oxidative stress during cryopreservation (Al-Mutary et al., 2020; Souza et al., 2019).

Glutathione, a key non-enzymatic antioxidant present in both reduced ((l-γ-glutamyl-l-cysteinyl-glycine: GSH) and oxidized (GSSG) forms, plays a vital role in repairing thiol groups (-SH) in proteins damaged by oxidative stress. Additionally, it protects the cellular membranes by preventing LPO and subsequent ROS generation (Lenzi et al., 1994). Widely being distributed in living cells, GSH serves as a vital biological antioxidant, participating in intracellular defense mechanisms against oxidative damage (Adeoye et al., 2018). Recent studies have demonstrated that supplementation of GSH in semen from various farm animals, including bulls, goats, pigs, dogs, and roosters, reduces cryodamage and improves the quality of cryopreserved spermatozoa (Ahmad et al., 2021; Andersen et al., 2018; Das et al., 2021; Estrada et al., 2014; Masoudi et al., 2018; Ogata et al., 2022a).

On the other hand, l-ascorbic acid (AA) is considered one of the major antioxidant constituents of seminal plasma (Walczak–Jedrzejowska et al., 2013). AA naturally occurs in seminal plasma, playing a crucial role in scavenging free radicals and facilitating various mechanisms that inhibit oxidative processes such as LPO (Anane & Creppy, 2001). As the primary water-soluble antioxidant in plasma, AA serves as a co-factor for at least eight enzymes in vivo and exerts antioxidant effects through its interaction with ROS (Michael et al., 2008). Incorporation of AA into extenders holds promise for enhancing sperm quality by mitigating cell damage through its ongoing radical-neutralizing activity. Notably, the antioxidant function of AA involves the inhibition of LPO induced by Fe^2+^ and Cu^+^ ions (Halliwell & Gutteridge, 2015). Various studies have demonstrated promising results in improving semen quality by supplementing semen extenders with AA (Alavi, 2014; Bucak et al., 2008; Gangwar et al., 2016; Mamouei et al., 2021; Pahlevy et al., 2022; Saputro et al., 2022; Uysal & Bucak, 2007).

Although individual supplementation of GSH and AA in semen extenders resulted in promising improvements in post-thaw sperm quality, no previous study has either directly studied their effects in the same study or evaluated the potential synergistic effects of their combined use in buck semen cryopreservation. Therefore, the objective of this study was to evaluate the individual and combined effects of GSH and AA on the quality and fertility of cryopreserved Black Bengal buck spermatozoa.

## 2 Materials and methods

### 2.1 Chemicals

Unless stated specially, all reagents and chemicals used in this study were procured from Sigma-Aldrich (St. Louis, MO, USA), Thermo Fisher Scientific (Waltham, MA, USA), and Sisco Research Laboratories Pvt. Ltd. (Mumbai, India).

### 2.2 Ethical approval

All the experimental procedures were conducted under the administrative supervision of the Bangladesh Agricultural University Research System (BAURES) and were approved by its Ethical Standard of Research Committee (ESRC/114/AH/2025).

### 2.3 Experimental site and animal management

Five mature Black Bengal bucks (1.9 to 2.5 years old) with good health and strong sexual desire were selected for this study. These bucks were maintained at the Goat Research Farm under the Goat Production Research Division of the Bangladesh Livestock Research Institute (BLRI), Dhaka, Bangladesh (23°53′19.201″N, 90°16′25.669″E). The bucks were reared in a semi-intensive system which included stall-fed conditions and an open space in front of the shed for movement. The bucks received 300 g of commercial concentrate in mash form, 40 g of germinated gram in the morning, and green grass was provided ad libitum. Clean and safe water was also provided ad libitum. Regular deworming (every 3 months) and vaccination against PPR and anthrax were performed on the bucks.

### 2.4 Semen collection and evaluation

Ejaculates (*n*= 30) were collected twice a week using an artificial vagina (AV) during the period from October 2023 to February 2024. Immediately after collection, the ejaculates were immersed in a warm water bath at 37 °C until assessment in the laboratory. Ejaculate volume was measured in mL from the graduated collection vial, and a hemocytometer was used to determine the concentration (10^6^/mL) of spermatozoa. Sperm motility, kinematics, and morphology were evaluated using a Computer-Assisted Sperm Analyzer (CASA) (Hamilton Thorne, IVOS II). Only ejaculates with >70 % motile sperm, 80 % normal morphology, and sperm concentrations >3000 × 10^6^/mL were selected for further processing (Seifi-Jamadi et al., 2016).

### 2.5 Extender preparation and freeze-thawing process

A commercial egg yolk-based extender, Triladyl**®** (Minitub GmbH, Tiefenbach, Germany), was used for the dilution of semen. A stock solution of the triladyl extender was prepared by combining 20 % triladyl solution, which contains glycerol, tris, citric acid, buffers, sugar, water, and antibiotics, with 20 % egg yolk and 60 % tris-citrate buffer. The prepared extender was thoroughly mixed to achieve homogeneity before further processing. After the initial evaluation, the ejaculates were pooled (*n*= 6; one per trial) at 37 °C to eliminate individual differences (Seifi-Jamadi et al., 2016) and subsequently diluted with triladyl extender to achieve a final sperm concentration of 200 × 10^6^/mL (Rezaei et al., 2023). After that, the semen samples were evaluated with respect to different parameters such as sperm motility, kinematics, and morphology and split into four aliquots according to four treatment groups, namely: a control group (no antioxidants), a GSH group (control + 1 mM GSH), an AA group (control + 8 mM AA), and a combined group (control + 1 mM GSH and 8 mM AA). After dilution, the semen samples were kept in a cold handling cabinet and equilibrated at 4–5 °C for 4 h. Following equilibration, the semen samples were loaded in 0.25 mL of French mini-straw (Minitub GmbH, Tiefenbach, Germany) and sealed using an automated machine (MPP Uno, Minitub GmbH, Tiefenbach, Germany). Finally, the filled straws were positioned horizontally at a distance of 4 cm above the liquid nitrogen surface for 15 min, plunged directly into liquid nitrogen (−196 °C) and stored in a cryogenic container until use. A total of 500 frozen semen straws were produced, and at least two straws from each treatment per trial were utilized for the assessment of semen quality, while the remaining straws were distributed across three districts (Mymensingh Sadar, Netrokona, and Kishoreganj) for AI in goats to evaluate post-thaw fertility of spermatozoa. Each sample was immediately assessed after thawing at 37 °C for 30 s for post-thaw sperm characteristics, namely sperm motility, kinematics, morphology, viability, plasma membrane integrity, malondialdehyde (MDA), and hydrogen peroxide (H_2_O_2_) status as follows:

### 2.6 Post thaw evaluation

#### 2.6.1 Sperm motility, kinematics, and morphology assessment

Sperm motility, kinematics, and morphology of cryopreserved semen were evaluated using a CASA (Hamilton Thorne, IVOS II). The CASA settings were as follows: temperature 37 °C; frame rate, 60 Hz; frame count, 30; minimum contrast, 35; minimum cell size, 5 pixels; average cell size, 9 pixels; cell intensity, 110 pixels; average path velocity (VAP), 50 μm/s; straightness (STR), 70 %; VSL cut-off, 15 μm/s; and VAP cut-off, 30 μm/s (Miraz et al., 2022). For analysis, 5 µL of each prepared semen sample was placed on a prewarmed microscope slide, covered with a standard coverslip, and analyzed for sperm motility, kinematics, and morphology parameters. The following parameters were tested: total motile ( %), progressive motile ( %), slow ( %), bent tail ( %), coiled tail ( %), distal droplet ( %), distal midpiece reflex (DMR) ( %), proximal droplet ( %), normal fraction ( %), average path velocity (VAP μm/s), curvilinear velocity (VCL µm/s), straight-line velocity (VSL µm/s), trajectory linearity (LIN %), trajectory straightness (STR %), amplitude of lateral head movement (ALH µm), beat cross frequency (BCF Hz), and the wobble coefficient (WOB %).

#### 2.6.2 Sperm viability

To evaluate sperm viability, the eosin-nigrosin staining technique was employed as described by Das et al. (2021). Briefly, a small aliquot of semen was gently mixed with the stain, spread thinly on a microscope slide, air dried, and examined under a phase-contrast microscope (CKX41, Olympus, Tokyo, Japan) at 40 × magnification. Dead spermatozoa absorbed eosin stain partly or entirely and appeared pink, and live spermatozoa remained unstained. The percentage of live spermatozoa was calculated by examining a minimum of 200 cells across several fields under the microscope.

#### 2.6.3 Sperm membrane integrity

The hypo-osmotic swelling test (HOST) was conducted to determine the functional integrity of the sperm plasma membrane according to Ahmad et al. (2021) with slight modifications. Briefly, 50 µL of each semen sample was mixed with 500 µL of hypo-osmotic solution (190 mOsm/L: 0.735 g of tri-sodium citrate dihydrate and 1.351 g D (-) fructose per 100 mL of de-ionized water) and incubated at 37 °C for 45 min. Following incubation, a small aliquot of semen was placed on a clean glass slide, covered with a cover slip, and examined under a phase-contrast microscope at 40 × magnification (CKX41, Olympus, Tokyo, Japan). Spermatozoa with coiled or bent tails were considered to have functionally intact plasma membranes. The percentage of membrane-intact spermatozoa was determined by examining a minimum of 200 cells per sample.

#### 2.6.4 Sperm H₂O₂ status

H₂O₂ was assayed according to the method described by Yu et al. (2003) with slight modifications. Briefly, the semen samples were centrifuged (1500 × *g* for 15 min) to isolate the sperm pellet and resuspended in 2 mL of phosphate-buffered saline (PBS; pH 7.2) to achieve a sperm concentration of 200 × 10^6^/mL (Bucak et al., 2009). After that, 1 mL of the prepared sperm suspension was mixed with 2 mL of 0.15 % TiCl4 in 20 % H_2_SO_4_ (v/v) and kept at room temperature for 10 min. The mixture was subsequently centrifuged at 11,500 g for 12 min, and the absorbance was measured with a MULTISKAN™ SkyHigh Microplate Spectrophotometer (Thermo Scientific, Waltham, USA) at 410 nm. Finally, the H_2_O_2_ concentration was determined using the specific extinction coefficient (2.8 × 10^5^/mol/cm^3^) and expressed as nmol/mL.

#### 2.6.5 Sperm MDA concentration

The concentration of MDA in the sperm sample, an indicator for lipid peroxidation, was determined by thiobarbituric acid (TBA) reaction as described by Garg et al. (2009). Briefly, the semen samples were centrifuged (1500 × g for 15 min) to isolate the sperm pellet, which was subsequently resuspended in 2 mL of phosphate-buffered saline (PBS; pH 7.2) to achieve a sperm concentration of 200 × 10^6^/mL (Bucak et al., 2009). After that, 1 mL of the sperm suspension was mixed with 2 mL of TBA-trichloroacetic acid (TCA) solution (15 % w/v TCA, 0.375 % w/v TBA, and 0.25 N HCl). The mixture was heated in a boiling water bath at 100 °C for 15 min and then allowed to cool at room temperature. The mixture was subsequently centrifuged at 1200 g for 15 min, and the absorbance of the resulting supernatant was measured with a MULTISKAN™ SkyHigh Microplate Spectrophotometer (Thermo Scientific, Waltham, USA) at 535 nm. Finally, the MDA content was measured using the specific extinction coefficient (1.56 × 10^5^/mol/cm^3^) and expressed as nmol/mL.

#### 2.6.6 Non-return rate (NRR)

A total of 206 Black Bengal does were inseminated with frozen semen by trained AI technicians across three districts of Bangladesh (Mymensingh Sadar, Netrokona, and Kishoreganj). The does to be inseminated exhibited normal estrus cycles, and estrus was initially detected by the farmers based upon behavioral signs. Finally, the state of estrus was confirmed by trained AI technicians before performing AI. For the procedure, each doe was restrained with its hind legs upward. A sterilized and lubricated vaginal speculum was inserted through the vagina for visualization of the cervical opening under sunlight. A frozen–thawed semen straw was loaded into an AI gun fitted with a plastic sheath, gently inserted into the cervical os, and the semen was deposited slowly. The insemination was performed between 12 and 24 h after the onset of estrus (Susilowati et al., 2023). The pregnancy rate was reflected in the NRR (Ren et al., 2018), and pregnancy was confirmed by the doe owner over the phone 42 d (two cycles) after insemination (Rimi et al., 2023). If the does were not in estrus after 42 d, they were considered pregnant. The formula to estimate NRR is as follows:

$$\text{NRR}\left(\%\right)=n_{\text{no...return},\;T}/n_{\text{served}}\times 100$$

Where, n_served_ ​ is the number of does served, and n_no-return, T_ is the number of does that do not return to estrus within window T (42 d).

### 2.7 Statistical analysis

The study was designed as a completely randomized design (CRD) with four treatment groups and repeated across six independent trials. The data were compiled, tabulated, and analyzed on the basis of the objectives of the study. Mean values and SEM (standard error of the means) are reported. The data were analyzed using the multivariate analysis of variance (MANOVA), followed by Duncan’s multiple range test for post hoc comparisons when significant differences (*P*< 0.05) were observed. The chi-square test was used to determine the association between antioxidant supplementation and the NRR, and pairwise comparisons were performed using logistic regression with the control group specified as the reference category to compare the NRR. Statistical analyses were performed using SAS software, version 9.4 (SAS Institute Inc., Cary, NC). Correlation analyses were conducted using the programming language R (R 4.3.1), and graphs were generated using GraphPad Prism software, version 10.2.3.

## 3 Results

### 3.1 Attributes of fresh semen

Table 1 presents the parameters of fresh semen collected from Black Bengal bucks, such as volume, concentration, motility and morphology parameters, presented as mean values with their SEM. Ejaculate volume was 0.78 ± 0.04 mL, and sperm concentration was 3478.62 ± 26.58 × 10^6^/mL. The percentage of total motile, progressive motile, and slow motile spermatozoa was 84.08 ± 0.92, 66.76 ± 1.03, and 0.14 ± 0.03, respectively. For morphological parameters, the percentage of normal spermatozoa was 84.01 ± 0.81. The incidence of spermatozoa with tail defects was characterized by a percentage of 1.14 ± 0.17 for bent tails, 0.26 ± 0.04 for coiled tails, and 2.97 ± 0.24 for distal droplets. In the case of midpiece defects of spermatozoa, the percentage of DMR was 0.60 ± 0.07, and the proximal droplet was 5.01 ± 0.21.

**Table 1:** Values of different parameters of fresh Black Bengal buck semen (*n*= 23).

Parameters	Mean ± SEM	Minimum	Maximum
Volume (mL)	0.78 ± 0.04	0.40	1.20
Concentration (10^6^/mL)	3478.62 ± 26.58	3190.36	3661.29
Total motile ( %)	84.08 ± 0.92	78.60	91.20
Progressive Motile ( %)	66.76 ± 1.03	58.20	75.40
Slow ( %)	0.14 ± 0.03	0.00	0.40
Bent tail ( %)	1.14 ± 0.17	0.10	4.00
Coiled tail ( %)	0.26 ± 0.04	0.00	0.70
Distal Droplet ( %)	2.97 ± 0.24	1.10	5.60
Distal Midpiece Reflex (DMR) ( %)	0.60 ± 0.07	0.10	1.30
Proximal Droplet ( %)	5.01 ± 0.21	2.90	6.60
Normal Fraction ( %)	84.01 ± 0.81	79.60	90.23

Total Motile = Progressive + non-progressive; *n* = total number of ejaculates; SEM = standard error of the mean.

### 3.2 Seminal attributes of pooled semen

Table 2 represents the pooled semen parameters of the experimental bucks with their mean values and SEM. The percentage of total motile, progressive motile, and slow motile spermatozoa was 84.08 ± 1.06, 57.62 ± 0.97, and 1.31 ± 0.26, respectively. For morphological parameters, the percentage of normal spermatozoa was 86.69 ± 1.10. The percentage of spermatozoa with bent tails, coiled tails, and distal droplets was 2.68 ± 0.37, 0.52 ± 0.09, and 1.54 ± 0.16, respectively. Regarding midpiece defects of spermatozoa, the percentage of DMR was 3.16 ± 0.31, and the proximal droplet was 3.48 ± 0.37.

**Table 2:** The characteristics of Black Bengal buck semen after pooling with respect to different parameters (*n*= 12).

Parameters	Mean ± SEM	Minimum	Maximum
Total motile ( %)	84.08 ± 1.06	77.90	89.80
Progressive Motile ( %)	57.62 ± 0.97	52.30	61.30
Slow ( %)	1.31 ± 0.26	0.10	3.20
Bent tail ( %)	2.68 ± 0.37	0.70	5.00
Coiled tail ( %)	0.52 ± 0.09	0.10	1.20
Distal Droplet ( %)	1.54 ± 0.16	0.50	2.30
Distal Midpiece Reflex (DMR) ( %)	3.16 ± 0.31	1.60	4.90
Proximal Droplet ( %)	3.48 ± 0.37	1.60	5.00
Normal Fraction ( %)	86.69 ± 1.10	79.30	90.70

Total Motile = Progressive + non-progressive; *n* = total number of observations; SEM = standard error of the mean.

### 3.3 Sperm motility, kinematics, and morphological features

Table 3 presents the CASA-derived post-thaw sperm motility and kinematic measurements. As shown in Table 3, supplementation of GSH, AA, and GSH+AA significantly (*P*< 0.05) improved sperm total motility, progressive motility, VSL, VCL, VAP, and BCF compared to the control group. The combined supplementation of GSH+AA in the semen extender exhibited the highest post-thaw sperm motility and kinematic parameters over other treatments (*P*< 0.05). The GSH and AA groups did not differ significantly, but both showed significantly higher values (*P*< 0.05) than the control. However, no significant (*P*> 0.05) effects were observed on parameters such as slow spermatozoa, STR, LIN, ALH, and WOB between the treated and control groups.

**Table 3:** Post-thaw sperm motility and kinematic parameters of Black Bengal buck semen diluted in triladyl extender supplemented with GSH, AA, and their combination (*n*= 12).

Parameters	Experimental groups				SEM	*P* value
	Control	GSH	AA	GSH+AA		
TM ( %)	44.09^a^	58.87^b^	55.66^b^	65.76^c^	1.157	<0.01
PM ( %)	25.83^a^	34.83^b^	37.28^b^	43.00^c^	1.012	<0.01
Slow ( %)	2.05	2.29	1.43	1.79	0.327	0.298
VAP (µm/s)	87.67^a^	97.29^b^	99.18^b^^,^^c^	100.18^c^	0.780	<0.01
VSL (µm/s)	75.59^a^	87.24^b^	88.76^b^^,^^c^	89.81^c^	0.763	<0.01
VCL (µm/s)	148.79^a^	162.24^b^	165.07^b^	168.84^c^	1.087	<0.01
STR ( %)	81.74	81.82	83.74	83.82	0.782	0.103
LIN ( %)	48.59	48.64	48.76	49.66	0.656	0.620
ALH (µm)	7.96	8.11	7.99	8.09	0.135	0.837
BCF (Hz)	28.44^a^	33.16^b^	32.23^b^	32.73^b^	0.353	<0.01
WOB ( %)	58.27	57.92	56.70	57.07	0.425	0.073

a,b,c Values in the same row with different superscripts differ significantly (*P* < 0.05). *n* = total number of observations; SEM: standard error of the mean; TM: total motility; PM: progressive motility; Slow: Slow motility; VAP: average path velocity; VCL: curvilinear velocity; VSL: straight line velocity; STR: trajectory straightness; LIN: trajectory linearity; ALH: amplitude of lateral head displacement; BCF: beat cross frequency; WOB: wobble coefficient.

Table 4 presents the post-thaw morphological features of spermatozoa. Results showed that supplementation of GSH, AA, and GSH+AA resulted in higher (*P*< 0.05) values of the normal fraction of spermatozoa than the control group. Moreover, antioxidant-treated groups resulted in lower (*P*< 0.05) values of distal droplet and bent tail in comparison with the control group. However, no statistical differences were observed in the DMR, proximal droplet, and coiled tail of spermatozoa between the treatment groups (*P*> 0.05).

**Table 4:** Post-thaw sperm morphological features of Black Bengal buck semen treated with GSH, AA, and their combination (*n*= 12).

Parameters	Experimental groups				SEM	*P* value
	Control	GSH	AA	GSH+AA		
Normal Fraction ( %)	75.81^a^	82.27^b^	80.79^b^	83.18^b^	0.846	<0.01
**Mid piece abnormalities**						
Distal mid-piece reflex (DMR) ( %)	5.26	3.97	3.95	4.10	0.393	0.066
Proximal droplet ( %)	5.88	5.02	5.34	4.73	0.343	0.118
**Tail abnormalities**						
Distal droplet ( %)	5.20^a^	3.76^b^	3.80^b^	3.12^b^	0.343	<0.01
Bent Tail ( %)	7.56^a^	4.62^b^	5.12^b^	3.78^b^	0.536	<0.01
Coiled Tail ( %)	1.69	0.85	1.04	1.30	0.236	0.082

a,b,c Values in the same row with different superscripts differ significantly (*P* < 0.05). *n* = total number of observations; SEM = standard error of the mean.

### 3.4 Correlation coefficients (r) between different parameters post-thaw spermatozoa

**Fig. 1:**
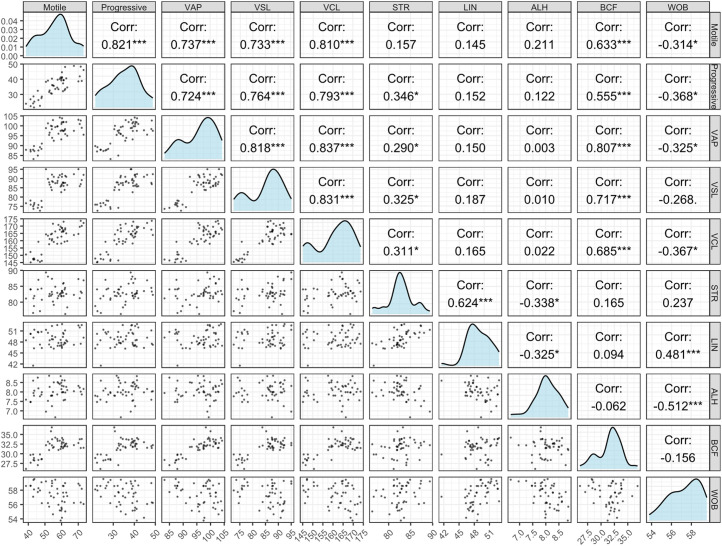
Pearson correlation coefficients with scatterplots and histograms among different parameters of post-thaw spermatozoa of Black Bengal buck. The diagonal shows the distribution density of each parameter. The lower panels represent pairwise scatterplots, while the upper panels display Pearson correlation coefficients (r) with significance levels (*P< 0.05, **P< 0.01, ***P< 0.001).

Fig. 1 represents the correlation coefficients between different parameters of post-thaw spermatozoa of Black Bengal buck. Total sperm motility showed a significant positive association (*P*< 0.05) with sperm kinematic variables such as VAP, VCL, VSL, and BCF. Moreover, sperm progressive motility significantly (*P*< 0.05) and positively correlated with VAP, VCL, VSL, and BCF. Sperm total motility also showed a strong significant positive association with progressive motility (*P*< 0.05). In addition, VAP, VSL, and VCL showed very strong positive associations (*P*< 0.05) among themselves. BCF exhibited significant positive correlations (*P*< 0.05) with motility, progressive motility, and velocity parameters, which indicate that increased sperm vigor is accompanied by higher beat frequency. In contrast, WOB and ALH were negatively correlated with LIN, STR, and BCF, implying that excessive lateral head displacement reduces the linearity and efficiency of sperm movement. Several parameters, such as LIN with motility or ALH with velocity traits, showed weak or negligible associations.

### 3.5 Sperm quality characteristics, MDA levels, and H_2_O_2_ status

**Fig. 2:**
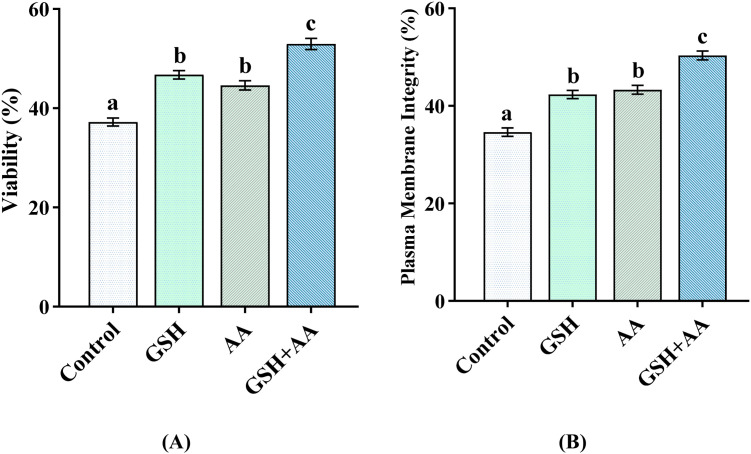
Effects of supplementation of GSH, AA, and their combination with semen extender on the viability (A) and plasma membrane integrity (B) of post-thaw spermatozoa of Black Bengal buck. Error bars indicate standard error of the mean. Bars with different letters (a, b, c) differ significantly (*P*< 0.05). Control: no antioxidants; GSH: 1 mM reduced glutathione; AA: 8 mM l-ascorbic acid; GSH+AA: 1 mM GSH+ 8 mM AA; *n*= 8).

Post-thaw sperm quality characteristics are presented in Fig. 2. The percentage of live and plasma membrane-intact cryopreserved spermatozoa was significantly higher (*P*< 0.05) for GSH+AA groups compared to other groups. In addition, significantly higher (*P*< 0.05) percentages of live and plasma membrane-intact spermatozoa were observed in GSH and AA groups relative to the control group. However, no statistical differences were observed in the percentage of live and plasma membrane-intact cryopreserved spermatozoa between the GSH and AA groups (*P*> 0.05).

**Fig. 3:**
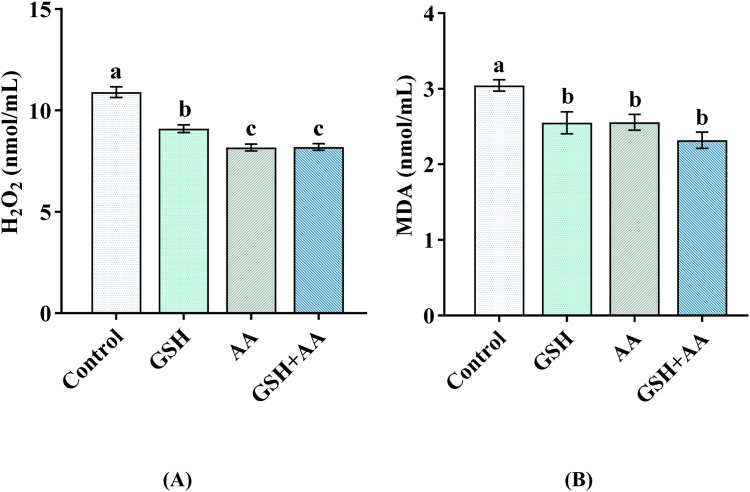
Effects of supplementation of GSH, AA, and their combination with semen extender on the H_2_O_2_ (A) and MDA (B) status of post-thaw spermatozoa of Black Bengal buck. Error bars indicate standard error of the mean. Bars with different letters (a, b, c) differ significantly (*P*< 0.05). Control: no antioxidants; GSH: 1 mM reduced glutathione; AA: 8 mM l-ascorbic acid; GSH+AA: 1 mM GSH+ 8 mM AA; H_2_O_2_ = hydrogen peroxide; MDA= malondialdehyde; *n*= 8).

Moreover, as shown in Fig. 3, the sperm MDA concentration and H_2_O_2_ levels in the GSH, AA, and GSH+AA supplemented groups were significantly lower (*P*< 0.05) in comparison with the control group. However, no significant differences (*P*> 0.05) were observed in the MDA concentration and H₂O₂ levels of spermatozoa between the antioxidant-treated groups except for GSH. The GSH-supplemented group showed a significant (*P*< 0.05) difference in H_2_O_2_ levels of spermatozoa compared to the AA, GSH+AA, and control groups (Fig. 3A).

### 3.6 *Non-return rate (NRR)*

In the evaluation of NRR, all antioxidant-supplemented groups exhibited significantly (*P*< 0.05) higher NRR than the untreated control group (Table 5). However, there is no significant difference (*P*> 0.05) between the three treatment groups. The combined group showed the highest NRR, with no statistically significant (*P*> 0.05) difference from GSH or AA alone.

**Table 5:** Effect of GSH, AA, and their combination on the sperm fertility of Black Bengal buck (*n*= 206).

Treatment Group	No. of does inseminated	No. of does not returned for further service	NRR ( %)
Control	44	17	38.64^a^
GSH	42	25	59.52^b^
AA	43	26	60.47^b^
GSH+AA	77	48	62.34^b^

a,b Values in the same column with different superscripts differ significantly (*P* < 0.05). *n* = total number of artificial inseminations.

Considering all the parameters studied, the addition of GSH, AA, and GSH+AA to the freezing medium could improve the quality and fertility of post-thaw Black Bengal buck spermatozoa.

## 4 Discussion

The cryopreservation process exposes spermatozoa to multiple stress factors, such as rapid thermal fluctuations, disruption of osmotic balance, crystallization of intracellular water, and oxidative damage. In general, the cryopreservation process leads to excessive ROS production and initiates multiple phases of cryodamage that adversely affect sperm quality and fertility (Len et al., 2019; Martin-Hidalgo et al., 2019). Incorporation of exogenous antioxidants into semen extenders has been demonstrated to effectively preserve sperm quality and enhance fertility potential in small ruminants during cryopreservation (Sharafi et al., 2015). In this study, the effects of the incorporation of GSH, AA, and their combination (GSH+AA) into semen extender on the quality and fertility of cryopreserved Black Bengal buck spermatozoa were investigated. Improvements were noted in sperm motility, kinematics, morphology, viability, plasma membrane integrity, the levels of oxidative stress markers (MDA and H_2_O_2_), and the fertility of cryopreserved spermatozoa. Notably, the group receiving both antioxidants exhibited superior sperm quality and fertility compared to all other groups.

Improvements in total and progressive motility, viability, and membrane integrity of post-thaw spermatozoa in the GSH and AA-treated groups are consistent with previous studies (Das et al., 2021; Zou et al., 2021), which reported similar improvements in goat semen quality following the addition of GSH and AA to the semen extender, respectively. Moreover, similar improvements in sperm quality due to GSH supplementation have been reported in bulls (Ogata et al., 2022b; Oikawa et al., 2018) and other animals (Andersen et al., 2018; Gangwar et al., 2018; Yeste et al., 2014). This effect may be attributed to the GSH-induced reduction in ROS and preservation of the structural and functional integrity of spermatozoa (El-kon & Darwish, 2011; Lanzafame et al., 2009). On the other hand, the presence of AA in extracellular fluids contributes to various cellular mechanisms that protect cells from oxidative stress by the disruption of various free radical processes, including LPO (Anane & Creppy, 2001). The addition of AA to the freezing extender provides significant protection against cryodamage in frozen-thawed semen by neutralizing ROS during cryopreservation. The findings of this study are consistent with several studies in bucks (Bodu et al., 2024; Mamouei et al., 2021; Saputro et al., 2022) and bulls (Motemani et al., 2017; Singh et al., 2020) that reported protective effects of AA at thawing. The beneficial effects of AA are primarily due to its potent antioxidant activity and ability to scavenge ROS, which are toxic metabolites of various metabolic processes (Dawson et al., 1992).

Motility is closely associated with sperm kinematics, generally, increased motility corresponds to increased VAP, VSL, VCL, STR, and LIN Santolaria et al. (2015). These kinematic parameters have also been suggested as potential reliable indicators of male fertility (Farooq et al., 2017) and are more sensitive to harmful effects of LPO than total motility (Ayad et al., 2017). In this study, the addition of GSH and AA to the semen extender significantly enhanced sperm kinematic parameters, including VAP, VSL, VCL, and BCF. These results align with previous findings, which reported significant enhancements in VAP, VSL, VCL, LIN, and BCF in Guanzhong dairy goats when GSH was added to the semen extender (Zou et al., 2021). This result may favor fertilization, as higher values of BCF and LIN facilitate sperm migration and penetration through the cervical mucus (Mortimer, 2000). Furthermore, CASA-derived parameters can serve as reliable fertility indicators for post-thaw goat spermatozoa, as samples that resulted in successful pregnancies following AI exhibited significantly higher mean BCF values compared to those that failed to achieve conception (Dorado et al., 2010). Similarly, research has shown that BCF together with VAP, VSL, VCL, LIN, and ALH are related to better sperm fertility in buffalo bulls and sheep (Del Olmo et al., 2013; Singh et al., 2017).

This study also revealed improved values of the normal morphology of post-thaw spermatozoa supplemented with GSH and AA in freezing extenders. This might be due to the reason that the supplementation of GSH and AA may have neutralized the generation of ROS. The freeze-thaw process elevates ROS that peroxidize tail-membrane lipids and damage axonemal proteins, such as actin and tubulin, which leads to structural abnormalities, including bent and coiled tails (Wang et al., 2025; Zampini et al., 2020). By neutralizing ROS, GSH and AA mitigate this oxidative damage, thereby maintaining membrane and structural integrity. This finding is consistent with previous results, which reported the lowest percentage of abnormal spermatozoa in GSH-treated groups of Beetal buck semen compared with the control in cooled semen (Sarangi et al., 2017). The increased percentages of normal sperm morphology with AA supplementation in the semen extender are consistent with other researchers who reported improved normal morphology in bucks (Memon et al., 2013) and in rams (Azawi & Hussein, 2013). Moreover, the percentage of abnormal spermatozoa, such as distal droplets, was reduced significantly in the antioxidant-treated groups. Although distal cytoplasmic droplets generally indicate defective spermatogenesis, cryopreservation induces osmotic and oxidative stresses that can produce membrane blebs that are morphologically similar to distal droplets under routine microscopy. These blebs result from plasma membrane swelling and detachment from the actin cortex due to osmotic shock and ROS-mediated lipid and protein oxidation (González-Fernández et al., 2012; Li et al., 2021; Zampini et al., 2020). Antioxidants may mitigate these effects by preserving membrane–cortex integrity, thereby reducing secondary, cryo-induced, droplet-like features.

Oxidative stress plays a key role in sperm damage during cryopreservation, mainly through lipid peroxidation and the accumulation of ROS such as H₂O₂. In this study, the supplementation of semen extenders with GSH and AA significantly reduced the concentrations of MDA and H₂O₂ in frozen-thawed spermatozoa compared with those in the control group. These findings align with those of previous studies (Daramola & Adekunle, 2015; Zou et al., 2021), which revealed the protective roles of GSH and AA in reducing oxidative stress and preserving sperm quality in goat semen during freezing and thawing. GSH possesses strong antioxidant properties and effectively scavenges free radicals, both directly and by serving as a cofactor for glutathione peroxidase, an enzyme essential for reducing lipid hydroperoxides and H₂O₂ in spermatozoa. Moreover, GSH serves as a vital biological antioxidant, participating in intracellular defense mechanisms against oxidative damage (Adeoye et al., 2018). On the other hand, previous findings demonstrated that AA helps to mitigate ROS-induced damage and reduce MDA concentrations by interrupting free radical chain reactions (Fanaei et al., 2014).

NRR is an indirect but widely accepted indicator of fertility, and a lower NRR reflects a greater proportion of animals maintaining pregnancy following insemination. Supplementation of semen extender with GSH and AA increased the NRR in inseminated does, which indicates the improved fertilization capacity of spermatozoa. Collectively, these findings indicate that antioxidant supplementation improved the fertilizing ability of spermatozoa. This may be attributed to the fact that both GSH and AA are potent antioxidants that help mitigate oxidative stress during semen preservation, which is a major factor that compromises sperm motility, membrane integrity, and DNA stability. By scavenging ROS, these antioxidants maintain the structural and functional integrity of sperm, thereby increasing their ability to fertilize the oocyte and sustain early embryonic development. This finding concurred with a previous study, where in vitro fertilization experiment showed higher fertilization capacity of spermatozoa in the GSH-supplemented groups (Zou et al., 2021). The improvement in NRR aligns with lower MDA and H₂O₂ levels in our study, indicating that reduced oxidative stress supports sperm function and fertilizing capacity.

The results of our study revealed that sperm motility in post-thaw buck semen was positively correlated with VAP, VSL, VCL, STR, LIN, ALH, and BCF. This finding aligns with the results reported in a previous study, which demonstrated positive correlations among VAP, VSL, and BCF, as well as between VCL and VSL and between BCF and ALH in freezable Mithun semen (Perumal, 2014). Similarly, research has shown strong correlations between sperm progressive motility and velocity parameters in pigs and rabbits (Kwon et al., 2012; Lavara et al., 2005). These observations suggest a correlation and interrelation among sperm kinematic parameters.

To the best of our knowledge, research on combined doses of GSH and AA in bucks is quite limited, and this is the first study to report the synergistic effects of GSH and AA on goat semen cryopreservation. The combination of these two powerful antioxidants in our study worked synergistically to reduce oxidative damage and resulted in improved preservation of sperm parameters (Baumber et al., 2000). The synergistic effects of combined GSH and AA supplementation were also demonstrated in cryopreserved pig semen, with improvements in sperm motility, viability, and nuclear protein structure under H₂O₂-induced oxidative stress (Giaretta et al., 2015). Moreover, the association of GSH and AA in bovine semen was shown to effectively preserve spermatozoa across all evaluated parameters (Pinto et al., 2020). This effect may be attributed to the action of antioxidants on LPO that occurs during the cryopreservation process, and thus, this combination likely exerted a synergistic effect, with GSH acting on disulfide bridges owing to its thiol-reducing properties, and both GSH and AA acting on LPO (Giaretta et al., 2015). However, this study revealed that the association between GSH and AA resulted in better preservation of normal sperm morphology, such as the normal fraction, bent tail, and distal droplet. Contrary to our study, it was reported that the combined supplementation of GSH and AA has no effect on normal sperm morphology (Pinto et al., 2020). This variability in the results may be due to the differences in the concentrations of the antioxidants and the diluter experimentally used.

Overall, the results of this study support the potential of the supplementation of GSH and AA alone or in combination with semen extenders to mitigate cryoinjury and improve semen preservation.

## 5 Conclusion

The present study confirms that individual supplementation of GSH and AA to semen extender improves the quality and fertility of cryopreserved spermatozoa. Notably, combined supplementation with these two antioxidants resulted in even better improvements in sperm quality and fertility. Thus, the addition of GSH (1 mM), AA (8 mM), and their combination to semen extenders could serve as a potential strategy to enhance the fertility of spermatozoa and is suggested for use in Black Bengal buck semen cryopreservation.

## Ethical statement

1. All animal experiments were conducted in compliance with institutional, national, and international guidelines for the care and use of animals in research and were approved by the Bangladesh Agricultural University Research System (BAURES) Ethical Standard Research Committee (ESRC/114/AH/2025).
2. All authors have read and approved the final version of the manuscript submitted to Veterinary and Animal Science.
3. The work described is the authors’ original research.
4. The article has not been published previously.

## Data availability

All original data generated or analyzed during this study are included in the article and/or the supplementary material. Further enquiries can be directed to the corresponding author.

## CRediT authorship contribution statement

**Md. Abul Bashar:** Writing – original draft, Visualization, Methodology, Investigation, Formal analysis, Conceptualization. **Mst. Mahomudha Akhtar:** Writing – review & editing, Methodology, Formal analysis, Data curation. **Shahanaj Ferdousi Shejuty:** Writing – review & editing, Resources, Supervision. **Gautam Kumar Deb:** Writing – review & editing, Supervision, Resources. **Sadek Ahmed:** Writing – review & editing, Supervision, Resources. **M. A. M. Yahia Khandoker:** Writing – review & editing, Conceptualization, Supervision, Resources, Project administration, Funding acquisition.

## Declaration of competing interest

The authors confirm that they have no competing interests to disclose.
